# Sepsis: evolving concepts and challenges

**DOI:** 10.1590/1414-431X20198595

**Published:** 2019-04-15

**Authors:** R. Salomão, B.L. Ferreira, M.C. Salomão, S.S. Santos, L.C.P. Azevedo, M.K.C. Brunialti

**Affiliations:** 1 Disciplina de Infectologia, Escola Paulista de Medicina, Universidade Federal de São Paulo, São Paulo, SP, Brasil; 2 Departamento de Moléstias Infecciosas e Parasitárias Faculdade de Medicina, Universidade de São Paulo, São Paulo, SP, Brasil; 3 Unidade de Terapia Intensiva do Hospital Sírio Libanês, São Paulo, SP, Brasil

**Keywords:** Sepsis-3, Inflammatory response, Immunometabolism, qSOFA, SOFA

## Abstract

Sepsis remains a major cause of morbidity and mortality worldwide, with increased burden in low- and middle-resource settings. The role of the inflammatory response in the pathogenesis of the syndrome has supported the modern concept of sepsis. Nevertheless, a definition of sepsis and the criteria for its recognition is a continuous process, which reflects the growing knowledge of its mechanisms and the success and failure of diagnostic and therapeutic interventions. Here we review the evolving concepts of sepsis, from the “systemic inflammatory response syndrome triggered by infection” (Sepsis-1) to “a severe, potentially fatal, organic dysfunction caused by an inadequate or dysregulated host response to infection” (Sepsis-3). We focused in the pathophysiology behind the concept and the criteria for recognition and diagnosis of sepsis. A major challenge in evaluating the host response in sepsis is to characterize what is protective and what is harmful, and we discuss that, at least in part, the apparent dysregulated host response may be an effort to adapt to a hostile environment. The new criteria for recognition and diagnosis of sepsis were derived from robust databases, restricted, however, to developed countries. Since then, the criteria have been supported in different clinical settings and in different economic and epidemiological contexts, but still raise discussion regarding their use for the identification versus the prognostication of the septic patient. Clinicians should not be restricted to definition criteria when evaluating patients with infection and should wisely use the broad array of information obtained by rigorous clinical observation.

## Introduction

The term sepsis comes from the Greek *σήψη*, which means putrefaction or putridity. It was characterized by Hippocrates as a dangerous, odoriferous, biological decay of the body (1).

For decades, sepsis was considered a systemic dissemination of infection leading to systemic clinical manifestations, involving the impairment of multiple organs and systems, with high morbidity and mortality. Septicemia and “*Blutvergiftung*” (the German word for sepsis that means blood poisoning) were the terms to define this condition. The understanding that systemic manifestations of infection could occur through inflammatory mediators without the need for microorganism dissemination led to the modern concept of sepsis (2).

According to a consensus meeting, published in 1992 and endorsed in 2003, sepsis was defined as the systemic inflammatory response syndrome (SIRS) caused by infection (3,4). Advances in understanding the pathogenic mechanisms of sepsis, the recognition that inflammatory and anti-inflammatory responses are triggered at the onset of infection, the involvement of other mechanisms in cellular and organic dysfunctions, the failure of intervention strategies targeting the inflammatory response, as well as the concern that the concept was very sensitive but lacked specificity, led to the review of the concept of sepsis in 2016 (5), which defined sepsis as a serious, potentially fatal, organic dysfunction caused by a dysregulated host response to infection and septic shock in a subset of patients in which underlying circulatory and cellular/metabolic abnormalities are sufficiently profound to substantially increase mortality (5). The new criteria as well as the implications in multiprofessional team training processes and intervention strategies are being debated by the scientific community (6,7).

Sepsis, as a manifestation of several endemic and epidemic diseases, has had a profound impact on the history of humankind. One of the most illustrative examples is the plague epidemic, which, in its septicemic form, decimated a third of the European population in the 14th century. Today, sepsis remains a major cause of morbidity and mortality worldwide. The actual number of cases is unknown, as there is limited information from developing countries. An extrapolation from high-income country data suggests global estimates of 31.5 million sepsis and 19.4 million cases, with a potential 5.3 million deaths (8). In a recent multicenter study performed in Brazil, one-third of intensive care beds were occupied by septic patients, with a mortality rate of 55.7% (9). Additionally, the rise in antimicrobial resistance and nosocomial sepsis has been a matter of concern, with studies suggesting that by the year 2050, 10 million people will have died annually worldwide due to healthcare-associated infections (10).

Despite the enormous progress of medical sciences and care, sepsis is still a challenge to define, recognize, and treat appropriately. In this paper, we will review the pathophysiological events underlying the concept of a dysregulated host response, emphasizing some changes that might in fact be an adaptation, and the challenges associated with the recognition and diagnosis of sepsis.

## The pathophysiology behind the concept

Sepsis was first described as the SIRS triggered by infection (3). The role of the inflammatory response in the pathogenesis of the syndrome had been established by clinical observation and supported by robust investigation. More than one hundred years ago, Willian Osler, an eminent clinician and educator, observed that “the patient appears to die from the body’s response to an infection rather than from the infection itself”, and decades ago, Otto Westphal, a prominent endotoxin researcher, wrote that “one of the most important fields of investigation is the search for mediators elicited by endotoxic signals and for the types of cells producing such highly active secondary products, with the hope to finally purify, identify, and even synthesize these biologically most interesting agents”. In a seminal experiment supporting the host response role in sepsis, Freudenberg and coworkers transferred macrophages from lipopolysaccharide (LPS)-sensitive mice (C3H/HeN) to LPS-resistant mice (C3H/HeJ) and rendered the latter susceptible to the lethal activity of LPS (11). Nevertheless, since the first sepsis consensus conference, our knowledge regarding the host-response mechanisms to pathogens and the tools available to investigate the complexity of this interaction have improved dramatically (12).

The pathogenesis of sepsis is complex and involves multiple aspects of the interaction between the infecting microorganisms and the host. The recognition of pathogens and the resulting cellular activation are fundamental for infection control. Paradoxically, the host inflammatory response is also the substrate for the pathophysiological changes in sepsis (12,13).

LPS and TLR4 are representatives of a concept conceived by Charles Janeway (14) that pathogen recognition is mediated by a set of receptors called pattern recognition receptors (PRRs) that detect common products of microorganism biosynthesis pathways (pathogen-associated molecular patterns, PAMPs). During sepsis, other PRRs and PAMPs are involved in the response to viral, fungal, and bacterial infections, including cell surface TLRs (TLR1, TLR2, TLR4, TLR5, and TLR6), where they recognize bacterial products such as lipoteichoic acid, lipoproteins, and flagellin, and intracellular TLRs (TLR3, TLR7, and TLR9) involved in the detection of genetic material from viruses and bacteria. Other receptors that recognize microbial products include nucleotide and oligomerization domain receptors (NLRs), retinoic acid-inducible gene I (RIG-I)-like receptors (RLRs), and lectin receptors of type C. Activation of these various receptors during infection is fundamental for the recognition of a wide range of microorganisms and result in complementary, synergistic, or antagonistic effects, thus modulating innate and adaptive immunity. It is important to note that PRRs may also recognize host products, termed alarmins or danger signals, such as heat shock proteins and high mobility group Box 1 protein (HMGB1), which play an important role in regulating the inflammatory response. The cascades of intracellular signaling and crosstalk of the PAMP and DAMP pathways have been reviewed elsewhere (12,15).

**Figure 1:**
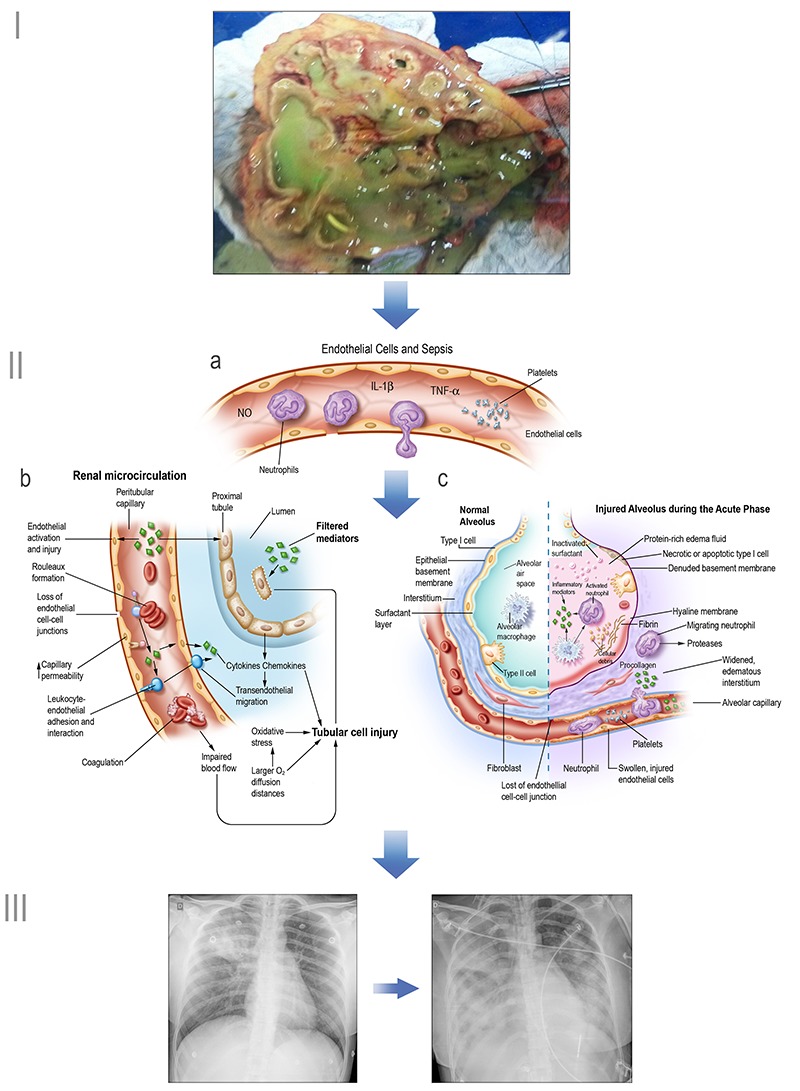
Sequential steps of sepsis pathogenesis and organ dysfunction. **I**, Infection: pyelonephritis in a patient with septic shock. **II,** Sepsis-induced endothelial changes leading to organ dysfunction; **IIa**, Schematic endothelial changes; **IIb**, Renal microcirculation: sepsis-induced injury to the endothelium, microcirculation and tubular cells. Adapted with permission from Springer-Nature (89) Copyright 2018; **IIc,** Alveolus-capillary changes during the acute phase of acute lung injury and acute respiratory distress syndrome. From (90) Copyright 2000. Reprinted with permission from Massachusetts Medical Society. **III**, Organ dysfunction: radiographic findings in a septic patient with progressive lung injury and acute respiratory distress syndrome.

The release of inflammatory mediators by innate immune cells upon pathogen recognition, such as tumor necrosis factor (TNF)-α, interleukin (IL)-6, and IL-1, and their effect on endothelial cells, resulting in the activation of coagulation, vasodilation, endothelial leakage, rolling and extravasation of neutrophils and inflammatory mediators to the extravascular space, underscores the pathophysiology of organ dysfunctions and hypotension during sepsis (16). Of paramount importance, the inflammatory response triggers procoagulant factors, while natural anticoagulant factors, such as activated protein C, anti-thrombin, and tissue factor inhibitors, are decreased in septic patients, resulting in a procoagulant state with multiple microthrombi and the obstruction of small vessels, which ultimately leads to intravascular disseminated coagulation (17). Figure 1 illustrates the sequential steps of infection, sepsis-induced endothelial changes, and organ dysfunction in a septic patient.

The inflammatory response triggered by infection should be finely regulated, and it is recognized that control mechanisms are triggered during sepsis. A compensatory anti-inflammatory response syndrome (CARS) was proposed to encompass these control mechanisms: a balanced response could result in infection control and recovery from organ dysfunction, as a predominance of the inflammatory response leads to organ dysfunction and death, while a predominance of the anti-inflammatory response, the so-called immunosuppression of sepsis, could lead to the persistence of foci of infection or the development of new secondary or even opportunistic infections and subsequent death (18). The timing of inflammatory and anti-inflammatory responses and their counter-regulatory mechanisms are central issues of current research (16,19 [Bibr B20]).

Evidence of a downregulation of cellular functions, mainly a decreased capacity to produce inflammatory cytokines, was observed in early experiments with peripheral blood cells from septic patients and gained renewed interest in recent years following the failure of clinical trials based on interventions in the inflammatory cascade (reviewed in ref. 20). Anti-TNF-α, recombinant-activated protein C and TLR4/MD2 antagonist clinical trials (21–23) illustrate the failure of these interventions over three consecutive decades. Studies evaluating the immune functions of peripheral blood cells have evidenced a broad impairment of the response: monocytes from septic patients have shown decreased HLA-DR expression and production of TNF-α and IL-6 after stimulation *in vitro*; neutrophils have been reported to present decreased chemotaxis, phagocytosis, and reactive oxygen species (ROS) generation, and importantly, decreased lymphocyte counts with reduced functionality are observed during sepsis (24–28). Thus, sepsis has emerged as an immunosuppressive condition (19), which predisposes the affected individual to secondary infection, commonly to agents with lower pathogenicity (29,30).

Previous studies evaluating inflammasome activation in critically ill and septic patients are representative of diverging results evaluating the state of inflammation or immunosuppression in sepsis. One study showed decreased NALP1 and CASP1 gene expression in septic shock patients compared with critically ill patients and healthy volunteers, supporting the conclusion that the changes in the inflammasome are part of the monocyte deactivation process that occurs in septic patients (31), while another study reported increased CASP1, IL-1β, and IL-18 expression in septic patients with acute respiratory distress syndrome (ARDS) compared with SIRS, supporting that the inflammasome pathway and its downstream cytokines play critical roles in ARDS development (32). We observed NLR upregulation (NLRP3 and NLRC4) and downregulation (NOD1 and NLRP1) in patients with sepsis, with more intense disturbances in non-survivors than in survivors (33).

Clearly, sepsis could no longer be solely characterized as a systemic inflammatory response triggered by an infection, and the revised concept – Sepsis 3 – has redefined sepsis as a life-threatening organ dysfunction caused by a dysregulated host response to infection (5). This concept has updated the complexity of the host response and consequent clinical presentation of the patients, opening avenues for new treatment strategies.

One important aspect that is not covered in this review and is overlooked in the Sepsis-3 concept is that the “dysregulated” host response may be driven by pathogen virulence factors. The emphasis that “what differentiates sepsis from infection is an aberrant or dysregulated host response and the presence of organ dysfunction” accurately reflects the role of host factors, such as gender, age, genetic backgrounds, and underlying diseases, but it underestimates the role of pathogen virulence factors in driving the host response and cellular dysfunctions. For instance, what drives commensal bacteria from being innocuous to being the causal agent of a potentially fatal infection? For example, *Staphylococcus aureus* is commonly found in our skin flora, but it is also one of the most prevalent pathogens in healthcare-associated infections, accounting for more than 11 thousand deaths per year in the USA (34). Disruption of the barrier defense, such as skin lesions or the presence of an invasive device, drifts the *S. aureus* from a commensal status to an invasive microorganism that starts producing biofilm (35). The production of leucocidins, such as Panton-Valentine leucocidin (PVL), and other virulence factors promotes neutrophil lysis and evasion of the immune system, dysregulating the host response and favoring the spread of the infection leading to sepsis (36). Moreover, the perception of an impaired immune state might be sensed by bacteria as an opportunity to invade and proliferate, becoming an opportunistic agent, a mechanism that could be present in secondary infections after a septic shock episode (37).

### Dysregulation versus adaptation

Different models were proposed to encompass the inflammatory response and immunosuppression in sepsis. The initial model was believed to be biphasic, that is, the inflammatory response would be followed by the immunosuppressive response (28). Later, it was recognized that both responses are concomitant, with one response prevailing over the other. However, two concepts emerged to support the pathogenesis of organ dysfunction and outcomes: one indicated that early deaths would result from the initial inflammatory response, which would prevail in the early stages of sepsis, and late deaths would result from new and opportunistic infections, secondary to the immunosuppressive status, which would prevail in protracted septic patients (19); the other, supported by transcriptomic studies, evidenced the persistence of the inflammatory response coupled with a compromised adaptive immunity during the course of the syndrome (38). These findings, coupled with clinical observations of persistent catabolism in long-term ICU patients, led to the proposal of persistent inflammation, immunosuppression, and catabolism syndrome (PICS) in patients who survive an initial sepsis or trauma event (39).

Interestingly, the above concepts converge to conclude that cells from the innate and adaptive immune system are, overall, hyporesponsive in protracted septic patients (19). This statement should be balanced, at least in part, by the argument that ongoing changes in cellular functions during sepsis include inhibited, preserved, and increased functions, and this modulation might be biologically relevant, aiming to control inflammation and preserve the anti-infective response (12).

The first point to be emphasized is that a downregulation of antigen presentation and production of inflammatory cytokines by monocytes from septic patients has been consistently observed in several studies as early as in admission samples, not only in protracted patient samples (24,40). One exception to these observations was our report of increased cytokine production by peripheral blood mononuclear cells (PBMCs) obtained from admission samples in a subset of septic patients without organ dysfunction, who were previously classified as having sepsis (41). Recovery of the capacity to produce inflammatory cytokines was reported in follow-up samples of septic patients (42), and in some reports, this recovery was associated with the survival outcome (24).

A second aspect is that downregulation is not a general phenomenon in innate immune cells during sepsis. Cavaillon and Adib-Conquy pointed out the similarities and biological significance of reprogramming cellular functions in LPS-tolerant monocytes and in sepsis (43). The biological activity of LPS may be modulated *in vivo* and *in vitro*, and the hypersensitivity and hyposensitivity might be induced under experimental conditions (reviewed in ref. 12). The hyporesponse to LPS, also known as tolerance, is induced by pre-exposure of cells and animals to small amounts of LPS or even other TLR agonists, e.g., cross-tolerance, which upon challenge with LPS blunt the production of inflammatory cytokines, such as TNF-α, and protect animals against an otherwise lethal injection of LPS (reviewed in refs. 12,43). Of note, increasing evidence shows that tolerance is not a hyporesponse but rather a modulation of cellular functions (44). Gene expression of LPS-tolerant monocytes shows a consistent modulation toward controlling inflammation (e.g., decreased expression of inflammatory cytokines, preserved IL-10 expression), disrupted activation of adaptive immunity, and preserved antimicrobial effectors (45). We found similar changes in TLR signaling pathway gene expression in a model of LPS-induced tolerance using human PBMCs (46) and in PBMCs from septic patients (12,47).

These results support the statement from Foster and coworkers that “genes encoding pro-inflammatory mediators should be transiently inactivated in tolerant macrophages to limit tissue damage. On the other hand, genes encoding antimicrobial effectors and other proteins that do not negatively affect tissue physiology should remain inducible even after repeated stimulation of TLRs to provide continuous protection from infection” (48). Accordingly, human monocytes that are tolerant to LPS present inhibited inflammatory cytokine production but retain the ability to phagocytose bacteria and to generate reactive oxygen species (45,49). In our study, CD163 and CD206 expression, markers of alternative activated macrophages (M2), did not correlate with tolerance and cytokine production in LPS-tolerant human monocytes (50).

Recent experimental work supporting a modulation rather than suppression of cellular functions in LPS tolerance showed that the cytokine response to LPS does not predict the host response to infection. In this work, pretreatment of mice with monophosphoryl lipid A (MPLA) significantly reduced LPS-elicited proinflammatory cytokines in plasma but improved the host response and survival to *Pseudomonas aeruginosa* infection (51). In bone marrow-derived macrophages, pretreatment with MPLA induced a persistent metabolic phenotype characterized by elevated glycolysis and oxidative metabolism as well as augmented phagocytosis and respiratory burst (51).

**Figure 2:**
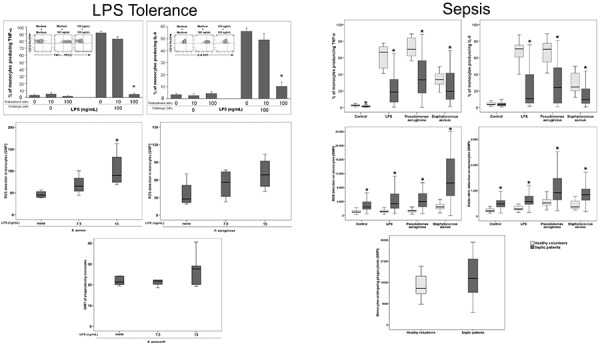
Similarities in monocyte functions observed in lipopolysaccharide (LPS)-induced tolerance and in septic patients evaluated at a cellular level by flow cytometry. Left panels represent modulated functions in LPS-tolerized monocytes: upper panels: reduced tumor necrosis factor (TNF)-α and interleukin (IL)-6 detection; middle panels: increased or preserved reactive oxygen species (ROS) generation; lower panel: preserved phagocytosis of *S. aureus.* Right panels represent modulated functions in monocytes from septic patients: upper panels: reduced TNF-α and IL-6 detection; middle panels: increased ROS and NO generation; lower panel: preserved phagocytosis of *E. coli*. Adapted from (42,49,50). Reproduction licenses available at creativecommons.org/licenses/by/4.0/.

We have previously argued that a similar modulation also takes place in human sepsis (12). In our studies, neutrophils obtained from septic patients presented with increased ROS generation and phagocytic activity (52). Furthermore, monocytes from septic patients that were hyporesponsive regarding the production of inflammatory cytokines (41) displayed an enhanced production of ROS and NO in response to LPS and gram-negative or gram-positive bacteria (52,53). These results have been confirmed and expanded recently in another cohort of septic patients, when we evaluated monocyte functions by flow cytometry in whole blood of septic patients and found preserved phagocytic activity, increased ROS and NO generation, and decreased production of inflammatory cytokines (42). Interestingly, most of the patients in this last cohort survived (88.2%), showing that this is a desirable reprogramming of cellular activities. In follow-up samples, e.g., after seven days of treatment, a trend toward a decrease in ROS and NO and an increase in cytokine production was observed, indicating a restoration of homeostasis (42). Similarities between LPS-tolerant human monocytes and monocytes from septic patients are shown in Figure 2.

**Figure 3:**
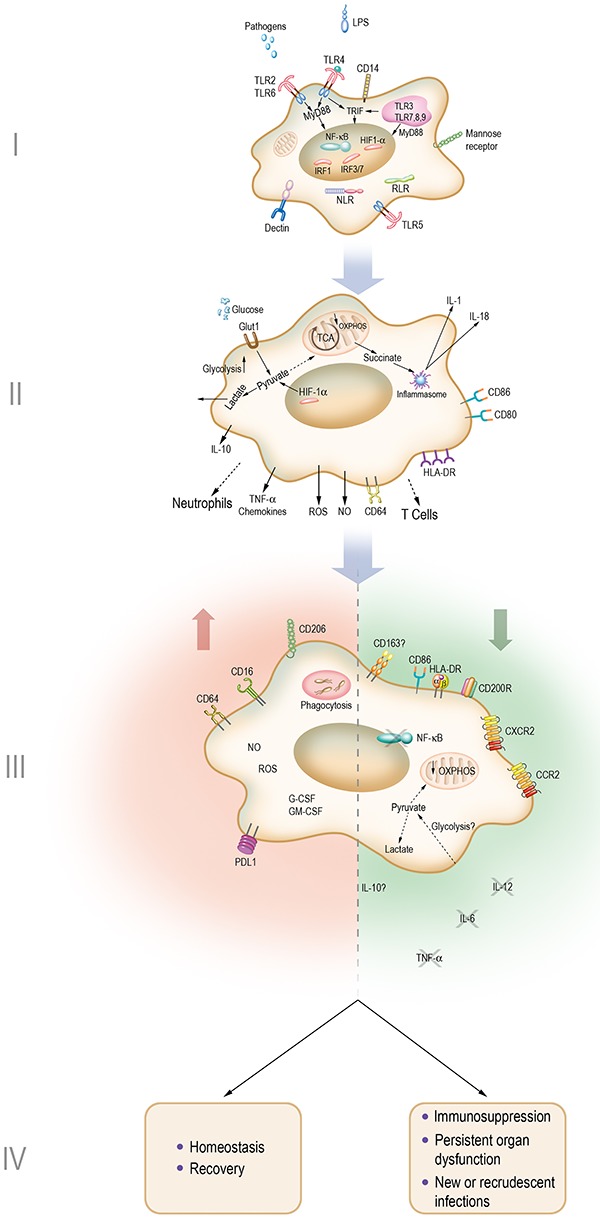
Modulation of monocytes/macrophages response during sepsis. **I**, In the initial infectious process, microorganisms and their products (pathogen-associated molecular patterns, such as lipopolysaccharide) are recognized by innate immune cells, such as macrophages through pattern recognition receptors, triggering intracellular inflammatory response pathways. **II**, Amplification of the inflammatory and immune response occurs through the synthesis of inflammatory mediators and cell-to-cell contact. Activated macrophages use glycolysis as a source of energy and biosynthetic intermediates to carry out its effectors' functions. **III**, Monocytes/macrophages reduce the production of inflammatory cytokines and the efficiency of T cell activation, while they retain the ability to phagocytose and kill microorganisms through the generation of ROS and nitric oxide (NO). **IV**, Resolution or immunosuppression. This phase represents the return to homeostasis and clinical recovery or, in a complicated course, the lack of an inflammatory response leads to immunosuppression, persistence of organ dysfunction, and emergence of new or recrudescent infections. Panel I adapted from (12) with permission. Promotional and commercial use of the material in print, digital or mobile device format is prohibited without the permission from the publisher Wolters Kluwer. Please contact permissions@lww.com for further information.

Is it possible to fit these findings to the observation of an increased incidence of infections caused by less pathogenic bacteria, viruses, and fungi in patients surviving sepsis? Or with progressing catabolic changes? As previously proposed, a model integrating these findings would be that, early in the infection process, an initial inflammatory response occurs when innate immune cells sense bacterial products and activate the adaptive immune response. In cases with an overwhelming inflammatory response, which may be due to host and bacterial factors, this initial response might be deleterious. In sequence, monocytes/macrophages would be reprogrammed to decrease the synthesis and release of inflammatory mediators and reduce antigen presentation and stimulatory accessory molecules, halting the amplification of the immune response while maintaining anti-infectious activity by preserving phagocytosis and the synthesis of ROS, NO, and antimicrobial peptides. This response would be effective during the course of an acute infection in a successfully treated patient, in which comorbidities, such as underlying diseases or severe trauma, do not impose continuous supportive therapy. In those patients who do not resolve the initial insult, both because of microorganism factors (e.g., bacterial challenge and resistance to antimicrobial agents) or host factors (e.g., immune deficiency or persistence of predisposing factors, such as patients in coma and under mechanical ventilation), the lack of inflammatory cytokines and activation of adaptive immunity will result in an ineffective response to control a persistent infection or will predispose the patient to a new infectious event, commonly by less pathogenic microorganisms, eliciting insidious clinical manifestations, as observed in more severe patients and those dying of sepsis (Figure 3).

### Metabolism and immunity

Mitochondrial dysfunctions and bioenergetic failure has long been recognized as an important pathophysiological mechanism underlying multiorgan dysfunction in septic patients (54), and an early mitochondrial biogenesis and antioxidant defense responses were related to the recovery and survival of those patients (55). Mitochondrial dysfunctions in sepsis occur by several mechanisms, including reversible inhibition of the electron transport chain complex and cytochrome c oxidase, oxidative inhibition of mitochondrial dehydrogenases and adenine nucleotide transporters, decreased cytochrome content, and respiratory uncoupling (reviewed in ref. (56)).

Mitochondrial dysfunction has also been shown to be induced by poly(ADP-polymerase)1 (PARP1, the major isoform of a family of poly(ADP-ribosyl)ation enzymes), a constitutive nuclear and mitochondrial enzyme that is a key regulator of DNA repair (57). Overactivation of PARP1 leads to NAD^+^ and ATP depletion and mitochondrial dysfunction. Pacher and colleagues demonstrated that PARP1-deficient mice show a reduced mortality rate in response to high doses of LPS (58), as well as in a model of septic shock induced by cecal ligation and puncture (59), and they propose that sepsis is a potential setting for the repurposing of PARP inhibitors for therapy in non-oncological diseases (60).

We were interested in evaluating the expression of genes belonging to the interacting TLR cascades, NADPH-oxidase, and mitochondrial oxidative phosphorylation in PBMC from septic patients. In a customized PCR array, we observed that genes related to mitochondrial oxidative phosphorylation from complexes I, IV, and V were downregulated, as were those involved in scavenging mtROS, such as super oxide dismutase (SOD)1 and SOD3, catalase, peroxiredoxin (PRDX)-3 and 4, and thioredoxin reductase (TXNDRD) 1 and 2. Accordingly, mitochondrial dysfunction and oxidative phosphorylation components were among the most altered canonical pathways in non-surviving patients (61).

Dysfunctional mitochondria in sepsis have led to two interesting concepts. MP Fink coined the term cytopathic hypoxia to characterize the cellular energetic derangement in sepsis not only from an impairment in oxygen delivery but also from an acquired intrinsic impairment in cellular respiration (reviewed in ref. (62)). Protty and Singer, considering that a reduction in energy consumption implies a reduction in cellular metabolism, proposed that the induction of a hypometabolic state resembling hibernation would protect the cells from dying once energy failure has developed (63).

There is increasing evidence that intracellular metabolic pathways modulate the function of immune cells, a field named immunometabolism (for a review see refs. (64–66)). Thus, mitochondria, which are essential metabolic and signaling organelles, have emerged as critical players in immune activation. As pointed out by Weinberg and coworkers “mitochondria not only sustain immune cell phenotypes but are also necessary for establishing immune cell phenotype and their function”. As examples, mitochondrial components activate the NLRP3 inflammasome and, through mitochondrial ROS and activation of HIF-1α, mediate LPS-induced inflammatory cytokines (66).

Several studies support glycolysis as a source of energy and modulation of immune functions. Some aspects of major interest for the immune modulation in sepsis will be briefly reviewed.

Under hypoxic conditions, cells will produce ATP by the breakdown of glucose via glycolysis, converting pyruvate to lactate rather than acetyl-CoA. Cells may preferentially use glycolysis for ATP generation, even when oxygen is not limiting, in a process known as aerobic glycolysis or the Warburg effect, which was first reported in cancer research (65). Glycolysis, in addition to rapid ATP generation, provides biosynthetic intermediates to support rapid cell growth and is, therefore, a preferred metabolic pathway in immune cells moving from the quiescent to the activated state (65). Enhanced glycolysis enables immune cells to generate sufficient ATP and biosynthetic intermediates to carry out their specific effector functions: in neutrophils, it feeds the pentose phosphate pathway and provides NADPH for key microbicidal pathways that are regulated by NADPH oxidase, and in macrophages supports phagocytosis and inflammatory cytokine production (64,65).

Hypoxia-inducible factor-1 (HIF-1a) is a key regulator of the metabolic commitment to glycolysis by promoting the expression of lactate dehydrogenase, which is responsible for the production of lactate from pyruvate, and of pyruvate dehydrogenase kinase (PDK), which inhibits pyruvate dehydrogenase, an enzyme complex that converts pyruvate to acetyl CoA (65). Our preliminary data examining the gene expression of HIF-1-related genes show reduced expression of EGLN2 and HIF1AN, inhibitors of HIF-1α, in septic patients, and increased PDK1 and HIF-1α in patients who did not survive (Ferreira BL et al., unpublished results).

In M1 macrophages, the tricarboxylic acid cycle is broken after citrate and after succinate, metabolites that can activate immune functions (64). Evaluating LPS-induced aerobic glycolysis in macrophages, Tannahil and coworkers demonstrated that succinate can serve as an activation signal in macrophages and promote IL-1β production by activating HIF1α (67). Citrate may be converted to itaconate, which has a direct antibacterial effect and an anti-inflammatory effect (68).

It has been shown that epigenetic reprogramming of myeloid cells by infection or vaccination confers nonspecific protection from secondary infections. This effect has been termed trained immunity and is dependent on changes in cell metabolism. Trained monocytes display a shift in metabolism with an increase in glycolysis that is dependent on the activation of mammalian target of rapamycin (mTOR) through a dectin-1/Akt/HIF1α pathway (69). Similarly, the TLR4 agonist MPLA drives broad resistance to infection via dynamic reprogramming of macrophage metabolism. Mice treated with MPLA have enhanced resistance to infection with *Staphylococcus aureus* and *Candida albicans.* In this condition, tissue macrophages exhibited increased phagocytosis and a respiratory burst that results in augmented microbial clearance and organ protection (70). Returning to the concept of modulation rather than suppression, these macrophages exhibit reduced TNF-α and IL-6 secretion in response to LPS. Again, blockade of mTOR signaling inhibits the development of the metabolic and functional macrophage phenotype and ablates MPLA-induced resistance to infection *in vivo*. Thus, HIF-1α and mTOR are key elements driving the metabolic pathway and immune modulation during the host response to infections (65).

In a well-designed study evaluating metabolic and immune changes in experimental and clinical sepsis, it was shown that whole blood leukocytes from patients with sepsis presented overexpression of genes encoding products involved in glycolysis and oxidative phosphorylation and evidenced a role for the mTOR-dependent HIF-1α axis in the regulation of leukocyte genes changes. In a subgroup of patients with monocytes lacking a cytokine response to LPS challenge (called immunotolerant), a generalized metabolic defect at the level of both glycolysis and oxidative metabolism has been reported (71), as illustrated in Figure 3.

## Recognition and diagnosis

As previously mentioned, the concept of sepsis changed from a SIRS caused by infection (Sepsis-1 and Sepsis-2) (3,4) to a life-threatening organ dysfunction caused by a dysregulated host response to infection (5). We have described above some relevant aspects of the immune response derangements, underscoring the dysregulated host response to infection. In this section, we will briefly discuss the impact of the operational definitions for the recognition and diagnosis of sepsis.

One of the greatest problems in the Sepsis-1 and Sepsis-2 definitions is the narrow limits in defining infection and sepsis. Indeed, SIRS criteria defining sepsis belong to the normal response to infection, such as pneumonia or urinary tract infection, and do not represent on their own a sign of a complicated course. Staging the syndrome with sepsis and severe sepsis would lead to the interpretation that sepsis could exist without severity. The term sepsis and severe sepsis are frequently used interchangeably; for instance, the Survival Sepsis Campaign published guidelines for the management of severe sepsis and septic shock (72). Thus, the new definitions encompassing infection, sepsis, and septic shock add clarity and, more importantly, stage the syndrome with increased severity, morbidity, and mortality.

According to Sepsis-3, sepsis is defined as severe, potentially fatal, organic dysfunction caused by an inadequate or dysregulated host response to infection. Thus, the term “severe sepsis” becomes redundant and should no longer be used. SIRS criteria (hyper- or hypothermia, tachycardia with HR >90 beats/min, tachypnea with respiratory rate ≥20 excursions/min, leukocytosis, or leukopenia) remain useful in recognizing the infectious process, even without organ dysfunction. The authors propose as operational criterion of organic dysfunction to define sepsis as a change ≥2 points in the Sequential [Sepsis-related] Organ Failure Assessment (SOFA) score (Table 1). Septic shock consists of a subgroup of septic patients, in whom circulatory and cellular/metabolic abnormalities are sufficiently important to substantially increase mortality. Operationally, septic shock represents sepsis requiring the use of vasopressor drugs to maintain a mean arterial pressure ≥65 mmHg and lactate >2 mmol/L (18 mg/dL), despite adequate volume replacement (5).

**Table 1:** Sequential organ failure assessment (SOFA) score. According to Sepsis-3, a variation in SOFA score ≥2 in a patient with suspected infection would be diagnosis of sepsis.

Organ system	SOFA 0	SOFA 1	SOFA 2	SOFA 3	SOFA 4
Respiratory(pO_2_/FiO_2_)	≥400	<400	<300	<200 (Mechanical ventilation)	<100 (Mechanical ventilation)
Hematologic(platelets x10^3^/mL)	≥150	<150	<100	<50	<20
Hepatic(bilirubin, mg/dL)	1.2	1.2-1.9	2.0-5.9	6.0-11.9	>12.0
Cardiovascular	MAP >70 mmHg	MAP <70 mmHg	Dopamine <5^a^ or dobutamine any dose	Dopamine <5.1-15, or adrenaline ≤0.1 or noradrenaline ≤0.1^a^	Dopamine >15, or adrenaline >0.1 or noradrenaline >0.1^a^
Neurologic(Glasgow coma scale)	15	13-14	10-12	6-9	<6
Renal(creatinine, mg/dL or urine output, mL/d)	≤1.2	1.2-1.9	2.0-3.4	3.5-4.9 Diuresis <500	>5.0 Diuresis <200

MAP: mean arterial pressure. ^a^Catecholamine doses are reported in mcg/kg per min for at least one hour.

As bedside criteria to identify adult patients with suspected infection outside the ICU who are likely to have poor outcomes, Sepsis-3 proposed the qSOFA (for quick SOFA): altered mentation, systolic blood pressure of 100 mm Hg or less, and respiratory rate of 22/min or greater. The presence of at least 2 variables suggests a patient at high risk for unfavorable outcomes, such as hospital death or a long-term ICU stay.

The Sepsis-3 definitions were welcomed overall, yet several questions rose immediately regarding operational aspects such as the absence of lactate as evidence of hypoperfusion to define sepsis while requiring elevated lactate to characterize septic shock. This raised the issue of the low sensitivity of the new criteria to recognize high-risk patients, which may delay interventions in countries or regions where mortality rates are already unacceptable. Thus, the Surviving Sepsis Campaign (SSC) and other associations, such as the Latin America Sepsis Institute (LASI), a nonprofit organization that provides training for hospitals and leads the SSC in Brazil, have acknowledged the conceptual advances of Sepsis-3 but have strengthened the relevance of SIRS as screening criteria and any organ dysfunction to define sepsis, including plasma lactate levels, as a parameter of metabolic dysfunction during sepsis and septic shock (7,73).

A major strength of the Sepsis-3 definitions is that the new diagnostic criteria were developed and validated in large retrospective databases from the USA and Germany (74,75), thus assuring the external validity of these criteria, at least in countries where the case-mix is similar to those evaluated. However, a similar validation should be performed in clinical settings that differ from those used to generate the new definitions and operational criteria, encompassing ICUs outside USA, patients presenting to the emergency service, and cohorts assisted in low- and middle-income countries.

Recent studies have addressed this important issue. In a multicenter study in Australia and New Zealand evaluating circa 180 thousand patients admitted to 182 ICUs, the discrimination of in-hospital mortality was significantly higher using SOFA (AUROC, 0.753 [99% CI, 0.750−0.757]) than either SIRS criteria (AUROC, 0.589 [99% CI, 0.585−0.593]) or qSOFA (AUROC, 0.607 [99% CI, 0.603−0.611]). The authors concluded that SIRS criteria provided no additional predictive use for mortality or a prolonged ICU stay beyond that achieved with SOFA and that the qSOFA score had little additional predictive value over the SIRS criteria among patients admitted to the ICU with suspected infection (76). The validity of the Sepsis-3 criteria was evaluated by Freund and coworkers in a prospective cohort of patients presenting to the emergency department with suspected infection. The performances of qSOFA and SOFA to predict in-hospital mortality (primary end-point) and admission to the ICU, length of ICU stay of more than 72 h, and a composite of death or ICU stay of more than 72 h (secondary end points) were compared with those of SIRS and the combination of SIRS and a blood lactate level greater than 2 mmol/L (18 mg/dL), a proxy of the former severe sepsis definition. The use of qSOFA resulted in greater prognostic accuracy for in-hospital mortality than did either SIRS or severe sepsis (77). These results suggest that qSOFA may be useful for the triage of patients with suspected infection and risk of unfavorable outcomes outside the ICU, while SOFA would have a better accuracy for those with sepsis who are already in the ICU (78).

The performance of Sepsis-3 criteria has also been evaluated in low- to middle-income countries. The prognostic value of the new definition of sepsis was evaluated in a single-center ICU showing increasing mortality along all three categories: infection with no organ dysfunction: 7/103 (7%); sepsis: 106/419 (25%); and septic shock: 198/435 (46%) (P<0.001); for Sepsis-2 definitions, ICU mortality differed only across the categories of severe sepsis [43/252 (17%)] and septic shock [250/572 (44%)] (P<0.001). Serum lactate improved the accuracy for values higher than 4 mmol/L in the no-dysfunction and septic shock groups (79). Similarly, SOFA and qSOFA were more sensitive and accurate than SIRS in predicting ICU and hospital mortality for critically ill cancer patients with suspected infection (80).

A recent study evaluated the performance of qSOFA and SIRS to predict excess hospital mortality in adults with suspected infection in low- and middle-income countries (LMIC). They included 9 cohorts encompassing circa 6.5 thousand patients with infection from 10 LMIC countries in Africa, Asia, and Central America. qSOFA discrimination was superior to SIRS (AUROC 0.70 [95% CI, 0.68−0.72] and 0.59 [95% CI, 0.57−0.62], respectively; P<0.001), thus supporting the use of qSOFA over SIRS in this context. There are two interesting aspects to be considered. The authors found that a moderate qSOFA, defined as qSOFA=1, was also associated with an increased risk of death, a finding that might help in triage and resource allocation; additionally, qSOFA performed well in infectious diseases such as dengue and malaria (81).

While the above studies support the use of qSOFA as a screening tool for disease severity, that is, to identify patients with infection who will likely have a worse outcome, some controversies persist regarding the sensitivity of the score. In a recent meta-analysis including two of the above commented studies (76,77), the sensitivity of the diagnosis of sepsis was consistently in favor of the SIRS criteria compared to qSOFA (1.32; 95% CI, 0.40−2.24; P<0.0001) (82). Data from the LASI database demonstrate that, among patients with infection and organ dysfunction (formerly severe sepsis), qSOFA was negative in 66%, suggesting that the use of this score to diagnose sepsis may be associated with missing almost two-thirds of patients with established organ failure. In addition, the mortality rate of qSOFA-negative patients in Brazilian public hospitals may reach up to 40%. As such, the use of a positive qSOFA ≥2 in scenarios of high mortality may fail to identify high-risk patients (Machado FR et al., unpublished data). Taken together, these results call for more studies evaluating the use of qSOFA to identify sepsis and a higher risk of death, especially in settings outside the ones used for validation of the definitions.

Despite the controversies, the Sepsis-3 criteria are supported by increasing evidence in different clinical settings and in different economic and epidemiological contexts. Clinicians and hospital teams should, however, not be restricted to the definition criteria when evaluating patients with suspected or unrecognized infection, which is indeed indicated in the consensus paper (5) and accompanying articles (74,75). SIRS, for instance, is useful for identification of an infected patient, and failure to meet 2 or more qSOFA criteria should not lead to a deferral of investigation or treatment of infection. Using SIRS criteria to identify patients with infection rather than label a patient with sepsis may help demand a more critical clinical evaluation of the patient, avoiding over-diagnosis and possible overload in laboratory exams and therapeutic interventions.

Lactate levels have been used for the screening and management of sepsis, and their wise application should continue to help physicians with clinical decisions. Lactate levels have been consistently associated with outcomes in sepsis (83,84). In the Sepsis-3 assessment of clinical criteria for sepsis, the addition of lactate levels to qSOFA statistically improved the predictive validity, but with little differences in identifying at-risk patients. Of note, for those with a qSOFA =1, the addition of higher lactate levels increased the identification of sepsis similar to those with 2 qSOFA points (74).

In the Sepsis-3 the assessment of new clinical criteria for septic shock, lactate levels were incorporated as a proxy for a cellular metabolic abnormality and as a variable independently associated with acute mortality. Here, the new definition diverges from previous widely used concepts because of the requirement for both the serum lactate level AND hypotension instead of either alone (OR) and by setting a lower serum lactate level cutoff of 2 *vs* 4 mmol/L, as currently used in the SSC definitions (75). While this discussion is far beyond this review, a few comments are presented for the clinical practitioner. What do you do if you do not have lactate measurements? Here, the “AND” should not change the clinical management and, as acknowledged in the new consensus paper, the use of a working diagnosis of septic shock using hypotension and other criteria consistent with tissue hypoperfusion may be necessary. Indeed, other parameters of hypoperfusion, such as urine output, mottling score, and capillary refill time, which are currently guiding the reassessment of fluid resuscitation, were not tested in the new definitions. The “OR” is important to detect hypoperfusion in normotensive patients, that is, cryptic shock, as pointed out by the SSC. An evaluation of lactate levels in the SSC database revealed that lactate values greater than 4 mmol/L increased mortality in an unadjusted regression analysis in non-hypotensive patients; lactate values greater than 4 mmol/L also significantly increased mortality combined with hypotension (85).

## Perspectives

At present, we are in a transition between the previous definition, which guided clinical management and supported successful interventions, and a new definition that has introduced unquestionable advances by incorporating current knowledge in sepsis pathophysiology and providing diagnostic criteria based on robust databases, but still raises discussion regarding the use of criteria for the identification versus the prognostication of the septic patient. In Brazil, the Latin American Sepsis Institute suggests that the hospital triggers the sepsis team based on SIRS and/or organ dysfunction, and they use qSOFA to identify patients with a high risk of death, tailoring resources and efforts according to the hospital characteristics.

In addition, advances in understanding of the mechanisms underlying the clinical course of sepsis have opened new perspectives for the identification of at-risk patients and for tailoring therapy. Transcriptomics and proteomics tools allowed us to envisage the magnitude of the host response to infection, the complexity of interactions and multiple biological processes, and the cellular functions that are disrupted during sepsis (38,86). Recently, these tools have enabled researchers to unravel the heterogeneity of patient responses and to characterize patterns or signatures that are related to sepsis outcomes. Davenport and coworkers, using transcriptomic analysis of peripheral blood leukocytes from septic patients admitted to the ICU, characterized two distinct sepsis response signatures (SRS1 and SRS2). The presence of SRS1 identified individuals with an immunosuppressed phenotype that included features of endotoxin tolerance, T-cell exhaustion, and downregulation of human leukocyte antigen (HLA) class II, and it was associated with higher mortality than SRS2 (87). In a similar approach, Scicluna and coworkers generated genome-wide blood gene expression profiles and characterized four molecular endotypes of sepsis, one of which (MARS-1) was found to be consistently associated with the worst outcomes (88). Stratifying patient risks based on the host response profile at the onset of a septic event might be a valuable tool for precision medicine.

Emerging new data will help us delineate a better algorithm for the identification and care of septic patients, both in the wide scope of well-organized and planed interventions as well as on an individual basis.
